# Oral health related quality of life and reasons for non‐dental attendance among patients with substance use disorders in withdrawal rehabilitation

**DOI:** 10.1002/cre2.476

**Published:** 2021-07-27

**Authors:** Anne Nordrehaug Åstrøm, Jorma Virtanen, Ferda Özkaya, Lars Thore Fadnes

**Affiliations:** ^1^ Department of Clinical Dentistry, Faculty of Medicine University of Bergen Bergen Norway; ^2^ Oral Health Centre of Expertise in Western Norway Hordaland Norway; ^3^ Department of Addiction Medicine Haukeland University Hospital Bergen Norway; ^4^ Department of Global Health and Primary Health Care University of Bergen Bergen Norway

**Keywords:** oral health related quality of life, substance us disorder, withdrawal rehabilitation

## Abstract

No study has assessed the socio‐behavioral distribution of oral health related quality of life (OHRQoL) among patients with substance use disorders receiving medically assisted rehabilitation therapy (MAR) in Norway.

**Objectives:**

To examine the prevalence of oral impacts on daily performances (OIDP) and its distribution among MAR patients in western Norway. We also examined whether oral impacts discriminate with different reasons for non‐dental attendance.

**Material and Methods:**

A cross‐sectional study focusing OHRQoL was nested to the INTRO‐HCV study and implemented in six rehabilitation clinics for people with substance use disorders. A total of 167 MAR patients completed personal interviews and oral clinical examination upon entering the clinic for their MAR medication.

**Results:**

The prevalence of oral impacts (OIDP > 0) was 61%. Logistic regression, adjusted for sex and age presented with odds ratios (OR) with 95% confidence intervals (CI) revealed that less than 20 remaining teeth (OR = 5.3 95% CI: 1.6–23.3) and dissatisfaction with dental care (OR = 5.1 95% CI: 1.3–19.0) increased the odds of having OIDP > 0. OIDP > 0 was also associated with insufficient dental follow‐up due to dental anxiety and poor experiences with perceived attitudes of dental workers. Means OIDP among people with negative experiences with attitudes of dental care workers were 3.1 (*SD* 0.8) compared to 1.4 (*SD* 0.7) among those without negative experiences, and 2.8 (*SD*) for those with dental anxiety compared to 1.8 (*SD*) among those without.

**Conclusion:**

OHRQoL among MAR patients was generally poor. To reach those with a need for dental care, modification of the existing rehabilitation approach toward closer collaboration between dental health care workers and others in contact with drug users might be necessary.

## 1 INTRODUCTION

In 2018, 269 million adults used illicit drugs and about 10% of them suffered substance use disorders, implying that their pattern of use is harmful, or they may experience drug dependence and or require treatment (United Nations, 2020; United Nations Office on Drugs and Crime, 2016). In Norway, use of illicit drugs, at least when it comes to cannabis, is concentrated mostly to younger adults, and the reported prevalence rates are generally higher among males than among females. In 2019, estimates of drug use last year among 16–34 year olds were 10.1, 2.1, 2.2 and 0.8% regarding cannabis, cocaine, MDMA and amphetamines, respectively (European Monitoring Center for drugs and drug addiction, 2019).

National and international studies have reported on negative health consequences of substance use, such as mental health problems, respiratory depression and overdose deaths, HIV/AIDS, tuberculosis and hepatitis (United Nations, 2020). In addition to these health problems, substance use has also been reported to give rise to a range of oral health related problems (Baghaie et al., 2017; Karlsen et al., 2017; Pedersen, 2016; Sheekarchizadeh et al., 2019; Shekarchizadeh et al., 2013a; Shekarchizadeh et al., 2013b; United Nations, 2020), directly and indirectly. Among individuals using substances, such as opiates, heroin, cocaine and amphetamine, tooth loss, progressive dental caries, bruxism, candida, mucosal dysplasia and periodontal disease are commonly observed and attributed to salivary hypo‐function and subsequent xerostomia (United Nations, 2020; United Nations Office on Drugs and Crime, 2016). Published reviews have shown that substance users suffer more frequent and serious oral diseases, than the general population, however, the frequency and severity appear to have a substantial range (Baghaie et al., 2017; Pedersen, 2016; United Nations, 2020; United Nations Office on Drugs and Crime, 2016). Rampant caries, involving the facial surfaces of anterior teeth and thus resembling early childhood caries has been observed among methamphetamine users (Pedersen, 2016). In addition to the drug use itself, inadequate oral hygiene, frequent intake of sugared products and alcohol, smoking together with insufficient dental care may contribute to development of oral diseases in individuals with substance use disorders (Shekarchizadeh et al., 2013a). Problems with dental care avoidance among drug abusers have been attributed to dental fear, as reported in several studies (Åstrøm et al., 2020; van Boekel et al., 2014). Further, we have found that dental health care professionals confirmed negative attitudes toward treatment of patients with substance use disorders and reported to be less familiar regarding oral health aspects of drug abuse (Åstrøm et al., 2020). In addition to poor lifestyle and environmental risk factors, medication utilized in the treatment of substance use disorders may have negative side effects on oral health (Sheekarchizadeh et al., 2019).

In Norway, the health‐ and well‐fare sectors have implemented major reforms for patients with substance use disorders (Helse og Omsorgsdepartementet, 2005, 2006, 2008; Helvig et al., 2017). As part of the rehabilitation of people with opioid dependence, about 8000 patients receive substitution therapy as part of MAR (Hjellum & Brukerplan, 2015). In principle, MAR patients are entitled to receive dental care free of charge, however, it is uncertain to which degree these groups have been reached optimally by dental health care services (Helvig et al., 2017; Sheridan et al., 2001a). Few studies have reported on oral health outcomes among people who have received prolonged treatment with opioid agonist therapy.

Evidence suggests that oral diseases have impact on the quality of life of individuals and populations (Locker & Slade, 1994). A previous study revealed that drug users were embarrassed about their mouth and teeth, and felt that the appearance of their smile affected their social life negatively (Shekarchizadeh et al., 2013a). A traditional normative approach to assess oral health status using only clinical disease indicators has serious inadequacies (Locker & Slade, 1994). Consequently, there is a need to incorporate patient reported outcomes in oral health studies (McGuire et al., 2014). Over time generic and disease specific oral health related quality of life (OHQoL) inventories have been developed to improve understanding of oral health impacts and complement traditional clinical oral disease measures (Adulyanon & Sheiham, 1997). Most studies of OHRQoL and its clinical and socio‐behavioral distribution have been conducted in the general populations (Gulcan et al., 2014; Holst & Dahl, 2008). It is unclear, however, whether findings from those studies generalize to populations with substance use disorder achieving medically assisted rehabilitation. Despite serious dental consequences of illicit drug use, few studies have examined the psychosocial impacts of oral diseases in illicit drug users and or the consequence of drug use on their quality of life (Antoniazzi et al., 2018; Karlsen et al., 2017; Marques et al., 2015; Mukherjee et al., 2018; Sharma et al., 2018). To the best of our knowledge, no study has assessed the socio‐behavioral and clinical distribution of OHRQoL of patients with substance use disorders who have received prolonged MAR in Norway.

This study aimed to examine the prevalence of OIDP and its distribution according to social, behavioral and clinical covariates focusing a group of patients receiving medically assisted rehabilitation in western Norway. We also examined whether oral impacts discriminate between patients with various degree of dental attendance and different reasons for non‐dental attendance.

## 2 MATRIALS AND METHODS

In 2017, an oral health study nested to the study *Integrated treatment of hepatitis C virus infection among people who inject drugs* (INTRO‐HCV; Fadnes et al., 2019), was conducted in six clinics providing treatment for people with substance use disorders in the municipality of Bergen, Western Norway. All patients with substance use disorders attending the clinics during the survey period (December 2017 to February 2018) were invited to participate in the study. A total of 167 patients (median 45 years, 77% male) completed structural interviews and clinical examination in the MAR clinic before picking up their MAR medication. Trained and calibrated oral health care personnel administered the interviews, counted the participants' number of remaining natural teeth and examined their oral mucosa. Both the interview and clinical examination were announced by information from staff in the clinics as well as information posters. To aid the clinical oral examination, we used a head‐lamp, white coat, gloves, face masks and disposable mouth mirrors for infection control. Participation was voluntary and the participants were presented with an informed consent. The present study was approved ethically by Regional Ethical Committee (no. 2017/2264).

### 2.1 Measures

The study outcome, OHRQoL/oral health related quality of life, was measured using the 8‐item oral impact on daily performances, OIDP frequency inventory (Adulyanon & Sheiham, 1997). We presented the following questions; “During the past six months how often have problems with your mouth and teeth caused you any difficulty with: (1) eating and enjoying food, (2) speaking and pronouncing clearly, (3) cleaning teeth, (4) sleeping and relaxing, (5) smiling and showing teeth without embarrassment, (6) maintaining usual emotional state, (7) enjoying contact with people and (8) carrying out major daily work.” Each OIDP item had originally the following response categories: “never affected,” “affected less than once a month,” “affected once or twice a month,” “affected once or twice a week” and “affected every day or nearly every day.” Each item was scored on a 5‐point Likert scale (1) never affected, (2) less than once a month, (3) once or twice a month, (4) once or twice a week, (5) every/nearly every day and dichotomized into (1) affected (including the original response categories [2–5] and [0] never affected including the original category 1). A sum frequency score was constructed in 2007 and 2012 from the eight dummy variables (range 0–8) and dichotomized into (0) no daily performance affected and (1) affected on at least one daily performance. The OIDP frequency inventory has demonstrated satisfactory cross‐sectional as well as longitudinal psychometric properties when applied in population‐based surveys in Norway and other Scandinavian countries (Karlsen et al., 2017; Sharma et al., 2018).

*Socio‐demographic characteristics* were measured in terms of age, sex, number of children below 18 years, and educational level. *Frequency use of illicit drugs* (*amphetamine*, *cannabis*, *heroin*) *during the past 30 days* was measured using a five graded scale ranging from (1) no days to (5) every day. *Satisfaction with oral health* (global measure) was measured by one question “How satisfied /dissatisfied are you with your teeth”‐ using a five‐graded response scale ranging from (1) very satisfied to (5) very dissatisfied. *Satisfaction with dental care* was assessed by question “How satisfied /dissatisfied are you with dental care received the last five years” using a five graded scale ranging from (1) very satisfied to (5) very dissatisfied. *Intake of sugared mineral water* was assessed by asking “How often do you take drinks containing sugars” using a five graded scale ranging from (1) more than once a day to (5) never. *Tooth brushing* was assessed by asking “How often do you usually brush your teeth” using the response scale (1) more than twice a day to (5) never or more seldom than once a week. Total number of remaining teeth was dichotomized for analysis into (1) 20 teeth or less, (2) >20 teeth.

### 2.2 Statistical analyses

Data were analyzed using SPSS version 25.0 (IBM Corp. released 2013, IBM Statistics for Windows, Amonk; IBM Corp.). We used chi‐square test in cross‐tabulation of OIDP with exposure variables. Internal consistency reliability of the eight item OIDP frequency score was assessed by Cronbach's alpha. Multiple variable logistic regression analyses with odds ratio (OR) and 95% confidence interval (CI) was used with OIDP as outcome variable and independent variables that revealed a statistical significant crude relationship in cross tabulation. General Linear Models (GLM) was used to assess the association between OIDP sum score and 8 categorical variables assessing perceived reasons for non‐dental attendance.

## 3 RESULTS

Table 1 depicts the profile of the study group in terms of socio‐demographic, behavioral and oral health related characteristics. A majority of the participants were men (77%) and a third were between 40 and 50 years of age. About half confirmed having completed secondary school education, while 42 and 38% used buprenorphine‐based and methadone‐based opioid agonist therapy, respectively. Totals of 24, 61 and 14% reported use of amphetamine, cannabis and heroin at least 1–3 days a week during the last 30 days. About half of the participants had 20 or less remaining teeth, 78% were dissatisfied with oral health and 61 and 42% reported at least one oral impact on daily performances and dental attendance at least once a year, respectively. The mean OIDP score amounted to 2.0 (*SD* 2.4) range (0–8).

**Table 1:** Frequency distribution of social, behavioral and oral health related factors in patients with substance use disorders receiving medically assisted rehabilitation

	Total
*Age categories*	% (n)
25	5.0 (8)
35	29 (48)
45	34 (57)
55	28 (47)
65	4 (7)
*Sex*	
Male	77 (126)
Female	23 (38)
*Number of children*	
No children	83 (138)
One or more	17 (29)
*Amphetamine*	
No use last 30 days	76 (95)
At least 1–3 days last 30 days	24 (30)
*Cannabis*	
No use last 30 days	39 (49)
At least 1–3 days	61 (76)
*Heroin*	
No use last 30 days	86 (14.4)
At least 1–3 days	14 (18)
*Completed secondary education or more*	
No	54 (72)
Yes	46 (62)
*Dental attendance*	
At least once a year	42 (70)
Less than annually	58 (96)
*Sugar‐sweetened mineral water*	
At least daily	61 (101)
Less than daily	40 (66)
*Tooth brushing*	
At least twice daily	52 (85)
Less than twice daily	48 (79)
*Number of teeth*	
20 and less	55 (57)
More than 20	45 (46)
*Oral quality of life*	
OIDP = 0	39 (60)
OIDP > 0	61 (92)
*Satisfied oral health*	
Satisfied	22 (36)
Dissatisfied	78 (130)
*Satisfied dental care*	
Satisfied	22 (36)
Dissatisfied	78 (130)

Table 2 depicts the frequency distribution of the eight OIDP items in the total study group and according to number of remaining teeth and satisfaction with oral health. The prevalence of oral impacts ranged from 44 to 15% with respect to difficulties with eating and sleeping/relaxing, respectively. Discriminative validity were demonstrated in that the single OIDP items as well as the sum‐OIDP score discriminated significantly between participants with and without 20 remaining teeth, and between participants satisfied and dissatisfied with oral health. Internal consistency reliability of the OIDP sum score in terms of Cronbach's alpha was 0.87.

**Table 2:** Criterion validity of OIDP. Frequency of various negative oral impacts by number of teeth and reported satisfaction with oral health

	20 teeth and less	Above 20 teeth	Unsatisfied oral health	Satisfied oral health	Total % (*n*)
Negative impacts on	% (*n*)	% (*n*)	% (*n*)	% (*n*)	
Eating	70 (37)	22 (11)**	52 (66)	19(7)**	44 (73)
Talking	42 (22)	10 (5)**	30 (39)	8 (3)**	26 (42)
Tooth cleaning	33 (17)	11 (5)*	26 (32)	11 (4)	23 (36)
Sleeping/ relaxing	21 (11)	10 (5)	17 (21)	11(4)	15 (25)
Smiling/showing teeth without feeling embarrassed	53 (27)	25 (12)**	43 (54)	17 (6)**	37 (60)
Stable feelings	34 (18)	8 (4)**	23(30)	11 (4)	21 (34)
Social relations	38 (20)	11 (5)**	29 (37)	6 (2)**	24 (39)
Daily work	36 (19)	6 (3)**	26 (33)	8 (3)*	22(36)
OIDP > 0	86 (43)	44 (20)**	68 (79)	36 (13)**	61 (92)

** *p* < 0.001,

* *p* < 0.05.

Table 3 depicts the prevalence of oral impacts (OIDP>O) according to socio‐demographic factors, type of drugs used during the last 30 days, oral health related behaviors, number of remaining teeth, satisfaction with oral health and satisfaction with dental care. Although not statistically significant, the prevalence of impacts was highest in younger MAR patients, males, those who attended a dentist more seldom than once a year and those reporting frequent sugar consumption. Prevalence of OIDP was significantly higher in patients reporting use of Cannabis 1–3 days a week, less than twice a day tooth brushing, having less than 20 remaining teeth, reporting dissatisfaction with oral health and dissatisfaction with dental care.

**Table 3:** Prevalence oral impacts (OIDP > 0) according to socio‐behavioral characteristics and oral health related indicators. Unadjusted % (*n*) and adjusted ordinary logistic regression analyses presented with odds ratios (OR) with 95% confidence intervals (CI)

	Unadjusted % (*n*)	Adjusted OR (CI)
*Age categories*		
25–45	61 (62)	1
>45	59 (30)	0.5 (0.1–2.1)
*Sex*		
Male	57 (65)	
Female	71 (25)	
*Number of children*		
None	58 (74)	
One or several	72 (18)	
*Use of illicit drugs last 30 days*		
Amphetamine (less than 1–3 days a week)	61 (53)	
Amphetamine (at least 1–3 days a week)	72 (18)	
Cannabis (less than 1–3 days a week)	52 (24)	1
Cannabis (at least 1–3 days a week)	71 (47)*	1.9 (0.5–6.8)
Heroin (less than 1–3 days a week)	62 (60)	
Heroin (at least 1–3 days a week)	73 (11)	
*Education/Completed higher*		
No	63 (41)	
Yes	64 (35)	
*Dental attendance*		
At least once a year	55 (36)	
More seldom	64 (56)	
*Sugared mineral water*		
At least daily	63 (58)	
Less than daily	57 (34)	
*Toothbrushing*		
At least twice daily	53(43)	1
Less than twice a day	69 (48)*	2.9 (0.9–10.9)
*Satisfied mouth and teeth?*		
Satisfied	36 (13)	1
Dissatisfied	68 (79)**	1.1 (0.2–5.0)
*Satisfied dentist*		
Satisfied	43 (35)	1
Dissatisfied	80 (53)**	5.1 (1.3–19.0)
*Number of remaining teeth*		
20 and less	86 (43)	1
>20	44 (20)**	5.3 (1.6–23.3)

* *p* < 0.01,

** *p* < 0.005.

In multiple variable ordinary logistic regression adjusted for age and sex (Table 3) and including covariates that were statistically significantly associated with oral impacts in unadjusted analysis, association with use of cannabis, tooth brushing frequency and satisfaction with oral health lost significance. The final adjusted model left number of remaining teeth (OR = 5.3 95% CI: 1.6–23) and satisfaction with dental care (OR = 5.1 95% CI: 1.3–19) the only statistically significant covariates of oral impacts on daily performances.

Table 4 depicts mean OIDP total scores according to perceived reasons for not having attended a dentist. The most frequently reported reasons for not having attended a dentist were in descending order; dental anxiety (42%), no felt need (23%), bad experience with dental care worker's attitude (20%), could not afford attending (18%), bad experience with treatment (14%), forgot meeting for appointment (9%), forgot scheduling appointment (9%) and did not have time (5%). General linear model‐ univariate, including all reasons for non‐attendance revealed statistically significant associations between OIDP sum score and “poor dentist attitude” and “dental anxiety,” only. Corresponding marginal means were 3.1 (*SD* 0.8) versus 1.4 (*SD* 0.7) and 2.8 (*SD* 0.8) versus 1.8 (*SD* 0.7). The model explained 12% of the total variance (adjusted *R*‐squared .123).

**Table 4:** Mean OIDP and standard deviation (*SD*) by reported reasons for not attending dental care

Reasons for not attending dental care	Mean OIDP (*SD*)
*No perceived need*	
Yes (23%, *n* = 38)	1.8 (0.9)
No (77%, *n* = 126)	2.7 (0.6)*
*Could not afford*	
Yes (18%, *n* = 30)	2.1 (0.8)
No (82%, *n* = 134)	2.4 (0.7)
*Did not have time (feeling too busy)*	
Yes (5%, *n* = 8)	2.7 (1.0)
No (95%, *n* = 156)	1.9 (0.6)
*Forgot scheduling appointment*	
Yes (9%, *n* = 14)	2.1 (0.9)
No (92%, *n* = 150)	2.2 (0.6)
*Forgot meeting for appointment*	
Yes (9%, *n* = 15)	2.4 (0.9)
No (91%, *n* = 149)	2.2 (0.7)
*Dental anxiety*	
Yes (41%, *n* = 68)	2.8 (0.8)
No (59%, *n* = 96)	1.8 (0.7)*
*Negative experiences with treatment*	
Yes (15%, *n* = 23)	1.9 (0.8)
No (85%, *n* = 131)	2.6 (0.7)
*Negative experiences with attitudes of dental care workers*	
Yes (20%, *n* = 32)	3.1 (0.8)
No (80%, *n* = 131)	1.4 (0.7)**

## 4 DISCUSSION

This study offers novel information by reporting influence of illicit drug use on oral health related quality of life in the context of socio‐behavioral and clinical covariates focusing patients with substance use disorders in withdrawal therapy in Norway. The findings revealed satisfactory psychometric properties of the OIDP inventory, in terms of criterion validity and internal consistency reliability for use among MAR patients. Thus, the prevalence of the total OIDP‐ and its separate eight oral impact scores varied in the expected direction with number of remaining teeth and reported satisfaction with oral health being highest in patients with less than 20 teeth and those dissatisfied with oral health. Internal consistency reliability in terms of Cronbach's alpha amounted to 0.88 which is above the recommended values of 0.70 and consistent with those reported in previous surveys (Adulyanon & Sheiham, 1997). This study suggests that MAR patients, aged 25–65 have poor OHRQoL that varies depending on the frequency of drug use, behavioral and oral health related characteristics. The prevalence of OIDP of 60% observed in this study deviates substantially from that of 18% estimated in the general Norwegian adult population (Astrom et al., 2005) as well as the prevalence of 50% estimated among younger adults (25–35 years) in Norway (Astrom et al., 2020). In accordance with the present findings, some previous studies have reported poorer health—and oral health related quality of life in drug addicts than in healthy controls as well as the general population (Antoniazzi et al., 2018; Karlsen et al., 2017; Marques et al., 2015; Mukherjee et al., 2018; Sharma et al., 2018).

Consistent with the addiction literature, this study revealed higher prevalence of oral impacts in frequent users of amphetamine, cannabis and heroin than in their counterparts reporting less frequent use. Previous studies have shown that exposure to cocaine, tobacco smoking and use of crack impact negatively on oral health related quality of life (Antoniazzi et al., 2018; Yazdanian et al., 2020). Lack of statistically significant association of OIDP with use of amphetamine and heroin may be explained by characteristics associated with the relatively young age profile of the study participants as well as their status as patients in withdrawal therapy. Norwegian MAR patients are offered free of charge access to some dental care and treatment services and this can play a role in changing their health‐ and oral health related attitudes and lifestyle (Shekarchizadeh et al., 2013b). Previous studies have shown that individuals agreeing to hospitalization for illicit drug dependence demonstrate concern about their health indicating some modifying effect of withdrawal therapy on the association between illicit drug use and oral health related quality of life (Barbadoro et al., 2008).

As shown in Table 3, the number of remaining teeth and satisfaction with dental care were the main predictors for oral impacts. Notably, the association of cannabis use with oral impacts disappeared in adjusted analysis, suggesting that its effect may be explained by oral hygiene behavior and dentition status. Number of remaining teeth associate strongly with illicit drug use and the association between cannabis use and oral impacts may thus be mediated by poor dentition status. A Brazilian study reported a similar finding in that the association of crack use with OHRQoL disappeared when clinical variables in terms of oral disease status were adjusted for in the multiple variable model (Antoniazzi et al., 2018).

Evidence suggests a strong association between irregular use of dental care and poor oral health (Riley & Gilbert, 2005). In contrast to findings in many studies of the general population, dental attendance did not associate significantly with OIDP among drug users in the present study. Nevertheless, participants' reason for non‐attendance associated with oral impacts. The most prevalent reason for not having attended a dentist was dental anxiety, no felt need and a poor attitude from dental health care personnel. Patients reporting dental anxiety and bad attitudes from dentists had poorer oral health related quality of life than their counterparts without these experiences. That unpleasant experience of dental care acts as barriers toward utilization has been demonstrated in previous studies of drug users as well as in the general population (Åstrøm et al., 2011; Sheridan et al., 2001b). Notably about 60% of the study group reported dental attendance less than once a year. This is below what is advised particularly when considering the high occurrence of oral diseases observed this group of patients with substance use disorders (Bernabe & Marcenes, 2010). Low prevalence rates of dental attendance has been reported among drug users also in other countries such as Iran and the United States with about 40–50% reporting their last dental visit more than 1 year ago (Shekarchizadeh et al., 2013b). Dental attendance rate observed among MAR patients in this study is less than in the general population of Norwegian adults where about 70–80% report attendance at least once a year (Astrom et al., 2018). Also in UK, drug addicts have been reported to be less likely to attend a dentist than are non‐drug users (Sheridan et al., 2001b).

This study has some limitations. Bias due to social desirability and recall bias may have occurred when assessing use of different drugs and possibly led to underestimation of the prevalence of drug use. Selection bias due to convenient sampling is possible as well as difficulties to recruit adequate numbers of drug exposed individual for optimal statistical power for some analyses. Another limitation is the incomplete information on some other co‐morbidities, which are also associated with oral health related quality of life in drug users.

## 5 CONCLUSION

OHRQoL among MAR patients was generally poor. The present results underscore the need for dental care strategies to improve OHRQoL among patients with substance use disorder. Patients need to be encouraged to use dental care available to them, and the services might need to be better tailored. To reach MAR patients with a need for dental care and ensure high quality and efficient dental care, modification of the existing rehabilitation approach toward closer collaboration between dental health care workers and others in contact with people with substance use disorders seems necessary. Such collaboration might enhance the uptake of dental health care services and thus improve oral health and quality of life among MAR patients in Norway.

## CONFLICT OF INTEREST

The authors declare no potential conflict of interest.

## AUTHOR CONTRIBUTIONS

**Anne Nordrehaug Åstrøma:** Conception and design, analysis and interpretation, drafting the work, final approval. **Lars Thore Fadnes:** Conception and design, revising the work critically for important intellectual content, contributing to analyses, final approval of the version to be published. **Jorma Virtanena:** Contribution to the final approval of the version to be published. **Ferda Özkayaa:** Contribution to the final approval of the version to be published.

## FUNDING STATEMENT

The study was funded by the University of Bergen. The nested INTRO‐HCV study was funded by The Norwegian Research Council (BEHANDLING, funding no 269855) and the Western Norway Regional Health Authority (“Åpen prosjektstøtte”) with the Department of Addiction Medicine, Haukeland University Hospital as the responsible institution. The funders had no role in the study design, data collection and analysis, decision to publish, or preparation of the manuscript.

## Data Availability

The data that support the findings of this study are available from the corresponding author upon reasonable request.

## References

[cre2476-bib-0001] Adulyanon, S. , & Sheiham, A. (1997). Oral impact on daily performances. In GD. Sade (Ed.), Measuring oral health and quality of life (pp. 151–160). University of North Carolina.

[cre2476-bib-0002] Antoniazzi, R. P. , Zanatta, F. B. , Ardenghi, T. M. , & Feldens, C. A. (2018). The use of crack and other illicit drugs impacts oral health related quality of life in Braziliens. Oral Diseases, 24(3), 482–488. 28949432 10.1111/odi.12786

[cre2476-bib-0003] Astrom, A. N. , Haugejorden, O. , Skaret, E. , Trovik, T. A. , & Klock, K. S. (2005). Oral impacts on daily performances in Norwegian adults: Validity, reliability and prevalence estimates. European Journal of Oral Sciences, 113, 289–296. 16048520 10.1111/j.1600-0722.2005.00225.x

[cre2476-bib-0004] Astrom, A. N. , Lie, S. A. , & Gulcan, F. (2018). Applying the theory of planned behavior to self‐reported dental attendance in Norwegian adults through structural equation modelling approach. BMC Oral Health, 18, 95. 29855371 10.1186/s12903-018-0558-7 PMC5984321

[cre2476-bib-0005] Åstrøm, A. N. , Ozkaya, F. , Virtanen, J. , & Fadnes, L. T. (2020). Dental health care workers' attitude towards patients with substance use disorders in medically rehabilitation (MAR). Acta Odontologica Scandinavica, 79(1), 31–36. 32449875 10.1080/00016357.2020.1769856

[cre2476-bib-0006] Åstrøm, A. N. , Skaret, E. , & Haugejorden, O. (2011). Dental anxiety and dental attendance among 15 year olds in Norway: Time trends from 1997 to 2007. BMC Oral Health, 11, 10. 21426538 10.1186/1472-6831-11-10 PMC3068991

[cre2476-bib-0007] Astrom, A. N. , Smith, O. R. , & Sulo, G. (2020). Early‐life course factors and oral health among young Norwegian adults. Community Dentistry and Oral Epidemiology, 113(4), 289–296. 10.1111/cdoe.12576 32918289

[cre2476-bib-0008] Baghaie, H. , Kisely, S. , Forbes, M. , Sawyer, E. , & Siskind, D. J. (2017). A systematic review and meta‐ analysis of the association between poor oral health and substance abuse. Addiction, 112(5), 765–779. 28299855 10.1111/add.13754

[cre2476-bib-0009] Barbadoro, P. , Lucrezi, D. , Propsero, E. , & Annino, I. (2008). Improvement of knowledge, attitude and behavior about oral health in a population of alcohol addicted persons. Alcohol and Alcoholism, 43, 347–350. 18326549 10.1093/alcalc/agn009

[cre2476-bib-0010] Bernabe, E. , & Marcenes, W. (2010). Periodontal disease and quality of life in Bristish adults. Journal of Clinical Periodontology, 37, 968–972. 20880054 10.1111/j.1600-051X.2010.01627.x

[cre2476-bib-0011] European Monitoring Center for drugs and drug addiction . (2019) Norway Country Drug Report. Retrieved from https://www.emcdda.europa.eu/countries/drug-reports/2019/norway_en

[cre2476-bib-0012] Fadnes, L. T. , Aas, C. F. , Vold, J. H. , Ohldieck, C. , Leiva, R. A. , Chalabianloo, F. , Skurtveit, S. , Lygren, O. J. , Dalgard, O. , Vickerman, P. , et al. (2019). Integrated treatment of hepatits C virus infection among people who inject drugs: Study protocol for a randomized controlled trial (INTTO‐HCV). BMC Infectious Diseases, 19, 943. 31703669 10.1186/s12879-019-4598-7 PMC6839172

[cre2476-bib-0013] Gulcan, F. , Nasir, E. , Ekback, G. , Ordell, S. , & Åstrøm, A. N. (2014). Change in Oral impacts on Daily Performances (OIDP) with increasing age: Testing the evaluative properties of the OIDP frequency inventory using prospective data from Norway and Sweden. BMC Oral Health, 14, 59. 24884798 10.1186/1472-6831-14-59 PMC4061514

[cre2476-bib-0014] Helse og Omsorgsdepartementet . (2005). Økte midler til tannbehandling for rusmiddelmisbrukere. Rundskriv 1‐12 2005. Oslo.

[cre2476-bib-0015] Helse og Omsorgsdepartementet . (2006) Utvidet fylkeskommunalt tannhelsetilbud. Rundskriv 1–2 2006. Oslo.

[cre2476-bib-0016] Helse og Omsorgsdepartementet . (2008). Vederlagsfri tannhelsetjeneste for personer under legemiddelassistert rehabilitering (LAR). Rundskriv 1–4 2008; Oslo.

[cre2476-bib-0017] Helvig, J. I. , Jensdottir, T. , & Storesund, T. (2017). Har gratis tannhelsetilbud til rusmiddelavhengige ført til forventet effekt? Den Norske Tannlaegeforenings Tidende, 127, 774–780.

[cre2476-bib-0018] Hjellum, K. , & Wåde‐ Engelsen, M. (2015). En kartlegging av kommunale tjenestemottakere over 18 år med psykiske problem, rusrelaterte problem eller samtidig rus og psykiske problem. NAPHA.

[cre2476-bib-0019] Holst, D. , & Dahl, K. E. (2008). Påvirker oral helse livskvalitet. Den Norske Tannlaegeforenings Tidende, 118, 212–218.

[cre2476-bib-0020] Karlsen, L. S. , Wang, N. J. , Jansson, H. , & Ansteinsson, V. (2017). Dental health and oral health related quality of life among selected drug abusers in Norway. Den Norske Tannlaegeforenings Tidende, 127, 316–321.

[cre2476-bib-0021] Karlsen, L. S. , Wang, N. J. , Jansson, H. , et al. (2017). Tannhelse og oral helserelatert livskvalitet hos et utvalg rusmiddelmisbrukere i Norge. Den Norske Tannlaegeforenings Tidende, 127, 316–321.

[cre2476-bib-0022] Locker, D. , & Slade, G. (1994). Association between clinical and subjective indicators of oral health status in older adults. Gerodontology, 11, 108–114. 7750964 10.1111/j.1741-2358.1994.tb00116.x

[cre2476-bib-0023] Marques, T. C. N. , Sarracini, K. L. , Cortellazzi, K. L. , Miahle, F. L. , de Castro, M. M. , Pereira, A. C. , & Ambosano, B. M. B. (2015). The impact of oral health conditions, socioeconomic status and use of specific substances on quality of life of addicted persons. BMC Oral Health, 15, 38. 25887243 10.1186/s12903-015-0016-8 PMC4382833

[cre2476-bib-0024] McGuire, M. K. , Scheyer, E. T. , & Gwaltney, C. (2014). Commentary: Incorporating patient‐reported outcomes in periodontal clinical trials. Journal of Periodontology, 85, 1313–1319. 25034790 10.1902/jop.2014.130693

[cre2476-bib-0025] Mukherjee, A. , Dye, B. , Clague, J. , Belin, T. R. , & Shetty, V. (2018). Methamphetamine use and oral health related quality of life. Quality of Life Research, 27, 3179–3190. 30076578 10.1007/s11136-018-1957-6 PMC8346633

[cre2476-bib-0026] Pedersen, A. M. L. (2016). Rusmidler og mundhulen. Aktuell Nordisk Odontologi., 41, 98–109.

[cre2476-bib-0027] Riley, J. L. , & Gilbert, G. H. (2005). Childhood dental history and adult dental attitudes and beliefs. International Dental Journal, 55, 142–150. 15997964 10.1111/j.1875-595x.2005.tb00311.x

[cre2476-bib-0028] Sharma, A. , Singh, H. , Mathur, A. , Aggarwal, V. P. , Gupta, N. , Makkar, D. K. , et al. (2018). Rout of drug abuse and its impact on oral health related quality of life among drug addicts. Addiction and Health, 10, 148–155. 31105912 10.22122/ahj.v10i3.567 PMC6511399

[cre2476-bib-0029] Sheekarchizadeh, H. , Khami, M. R. , Mohebbi, S. Z. , et al. (2019). Oral health status and its determinants among opiate dependents: A cross sectional study. BMC Oral Health, 19, 5. 30616605 10.1186/s12903-018-0691-3 PMC6323735

[cre2476-bib-0030] Shekarchizadeh, H. , Khami, M. R. , Mohebbi, S. Z. , et al. (2013a). Oral health of drug abusers: A review of health effects and care. Iranian Journal of Public Health., 42, 929–940. 26060654 PMC4453891

[cre2476-bib-0031] Shekarchizadeh, H. , Khami, M. R. , Mohebbi, S. Z. , et al. (2013b). Oral health behavior of drug addicts in withdrawal treatment. BMC Oral Health, 13, 11. 23368406 10.1186/1472-6831-13-11 PMC3583702

[cre2476-bib-0032] Sheridan, J. , Aggleton, M. , & Carson, T. (2001a). Dental health and access to dental treatment: A comparison of drug users and non‐drug users attending community pharmacies. British Dental Journal, 191, 453–457. 11720019 10.1038/sj.bdj.4801206

[cre2476-bib-0033] United Nations . (2020) Drug use and health consequences. World Drug Report. Retrieved from https://wdr.unodc.org/wdr2020/en/exsum.html

[cre2476-bib-0034] United Nations Office on Drugs and Crime . (2016). World drug report 2015. United Nations Publications.

[cre2476-bib-0035] van Boekel, L. C. , Brouwers, E. P. M. , van Weeghel, J. , et al. (2014). Health care professionals' regard towards working with patients with substance use disorders: Comparison of primary care, general psychiatry and specialist addiction services. Drug and Alcohol Dependence, 134, 92–98. 24099970 10.1016/j.drugalcdep.2013.09.012

[cre2476-bib-0036] Yazdanian, M. , Armoon, B. , Noroozi, A. , et al. (2020). Dental caries and periodontal disease among people who use drugs: A systematic review and meta‐analysis. BMC Oral Health, 20(1), 44. 32041585 10.1186/s12903-020-1010-3 PMC7011515

